# Two weeks of early time-restricted feeding (eTRF) improves skeletal muscle insulin and anabolic sensitivity in healthy men

**DOI:** 10.1093/ajcn/nqaa192

**Published:** 2020-07-30

**Authors:** Robert Jones, Pardeep Pabla, Joanne Mallinson, Aline Nixon, Tariq Taylor, Andrew Bennett, Kostas Tsintzas

**Affiliations:** MRC Versus Arthritis Centre for Musculoskeletal Ageing Research, School of Life Sciences, University of Nottingham Medical School, Nottingham, United Kingdom; MRC Versus Arthritis Centre for Musculoskeletal Ageing Research, School of Life Sciences, University of Nottingham Medical School, Nottingham, United Kingdom; MRC Versus Arthritis Centre for Musculoskeletal Ageing Research, School of Life Sciences, University of Nottingham Medical School, Nottingham, United Kingdom; MRC Versus Arthritis Centre for Musculoskeletal Ageing Research, School of Life Sciences, University of Nottingham Medical School, Nottingham, United Kingdom; MRC Versus Arthritis Centre for Musculoskeletal Ageing Research, School of Life Sciences, University of Nottingham Medical School, Nottingham, United Kingdom; School of Life Sciences, University of Nottingham Medical School, Nottingham, United Kingdom; MRC Versus Arthritis Centre for Musculoskeletal Ageing Research, School of Life Sciences, University of Nottingham Medical School, Nottingham, United Kingdom

**Keywords:** time-restricted feeding, free-living intervention, insulin sensitivity, skeletal muscle, energy balance and metabolism, body composition

## Abstract

**Background:**

Altering the temporal distribution of energy intake (EI) and introducing periods of intermittent fasting (IF) exert important metabolic effects. Restricting EI to earlier in the day [early time-restricted feeding (eTRF)] is a novel type of IF.

**Objectives:**

We assessed the chronic effects of eTRF compared with an energy-matched control on whole-body and skeletal muscle insulin and anabolic sensitivity.

**Methods:**

Sixteen healthy males (aged 23 ± 1 y; BMI 24.0 ± 0.6 kg·m^−2^) were assigned to 2 groups that underwent either 2 wk of eTRF (*n* = 8) or control/caloric restriction (CON:CR; *n* = 8) diet. The eTRF diet was consumed ad libitum and the intervention was conducted before the CON:CR, in which the diet was provided to match the reduction in EI and body weight observed in eTRF. During eTRF, daily EI was restricted to between 08:00 and 16:00, which prolonged the overnight fast by ∼5 h. The metabolic responses to a carbohydrate/protein drink were assessed pre- and post-interventions following a 12-h overnight fast.

**Results:**

When compared with CON:CR, eTRF improved whole-body insulin sensitivity [between-group difference (95% CI): 1.89 (0.18, 3.60); *P* = 0.03; η^2^_p_ = 0.29] and skeletal muscle uptake of glucose [between-group difference (95% CI): 4266 (261, 8270) μmol·min^−1^·kg^−1^·180 min; *P* = 0.04; η^2^_p_ = 0.31] and branched-chain amino acids (BCAAs) [between-group difference (95% CI): 266 (77, 455) nmol·min^−1^·kg^−1^·180 min; *P* = 0.01; η^2^_p_ = 0.44]. eTRF caused a reduction in EI (∼400 kcal·d^−1^) and weight loss (−1.04 ± 0.25 kg; *P* = 0.01) that was matched in CON:CR (−1.24 ± 0.35 kg; *P* = 0.01).

**Conclusions:**

Under free-living conditions, eTRF improves whole-body insulin sensitivity and increases skeletal muscle glucose and BCAA uptake. The metabolic benefits of eTRF are independent of its effects on weight loss and represent chronic adaptations rather than the effect of the last bout of overnight fast. This trial was registered at clinicaltrials.gov as NCT03969745.

## Introduction

Most nutritional health research focuses on altering the quantity and/or type of food consumed. Recent research suggests that the temporal distribution of nutrient intake (chrononutrition) can also play a role in mediating the health effects of a given diet. For instance, restricting the daily energy intake (EI) window to between 4 and 10 h, known as time-restricted feeding (TRF), elicits favorable metabolic effects in rodents independently of energy balance (1–3), which include protection against excessive body weight gain in response to high-fat and high-sucrose diets, reduced serum triglycerides, fasting insulin concentrations and hepatic fat content, and improved glucose tolerance. However, key differences in the adaptive metabolic response to fasting between rodents and humans, including rates of hepatic glycogen depletion (4, 5), may limit the translatability of these findings.

Studies investigating the effect of TRF in humans have primarily focused on adaptations to resistance training (6–8) and/or only examined fasting metabolism, energy balance, or weight loss (9–11). In a feasibility study, overweight individuals underwent 16 wk of TRF, during which they were required to reduce their daily eating duration to 10–12 h, resulting in significant weight loss and improved levels of self-reported sleep satisfaction and energy (12). Furthermore, improvements in glucose tolerance after TRF have been shown in adults who were overweight/obese (13–15). Short-term TRF has also been shown to reduce appetite markers and increase 24-h fat oxidation rates in overweight individuals (16). More recently, 12 wk of 10-h TRF in individuals with metabolic syndrome improved markers of cardiometabolic health, including blood pressure and circulating lipids (17). Currently, there is a paucity of research in this area using detailed metabolic measurements during the postprandial period, which comprises most of the waking day. In addition, no studies have examined the effect of TRF on skeletal muscle metabolism, which plays an important role in the disposal of an oral glucose load (18). Moreover, it is plausible that improved skeletal muscle anabolic sensitivity may underpin the preferential reductions in fat mass observed during TRF compared with an energy-matched control diet in humans undertaking resistance training (6).

An additional consideration is the optimal timing of the EI window. Diurnal variations in metabolic function, including glucose tolerance, were established several decades ago (19, 20). More recent, randomized crossover studies demonstrate favorable acute metabolic responses to a meal consumed earlier in the day (21, 22), and there is evidence suggesting chronically shifting a greater proportion of EI to earlier in the day may also be beneficial (23, 24). Early time-restricted feeding (eTRF) is a dietary strategy combining these approaches by shifting EI to earlier in the day and extending the length of the overnight fast. A recent supervised, controlled feeding randomized controlled trial found that 5 wk of eTRF improved whole-body insulin sensitivity in individuals with prediabetes independently of weight loss (15). However, metabolic responses were not compared after a matched duration of fast, and the effects of eTRF in a free-living setting have not yet been examined. This includes objectively measured physical activity (PA) levels and glycemic variability using continuous glucose monitors (CGMs).

The primary aim of this study was to compare the effects of 2 wk of free-living eTRF and an energy-matched (restricted) control intervention on markers of whole-body and skeletal muscle insulin and anabolic sensitivity in healthy young men. Secondary aims were to assess the changes in body composition and patterns of PA following 2 wk of intervention. It was hypothesized that, compared with the control, eTRF will confer favorable metabolic benefits during the postprandial period in healthy humans.

## Methods

### Ethical approval

**FIGURE 1:**
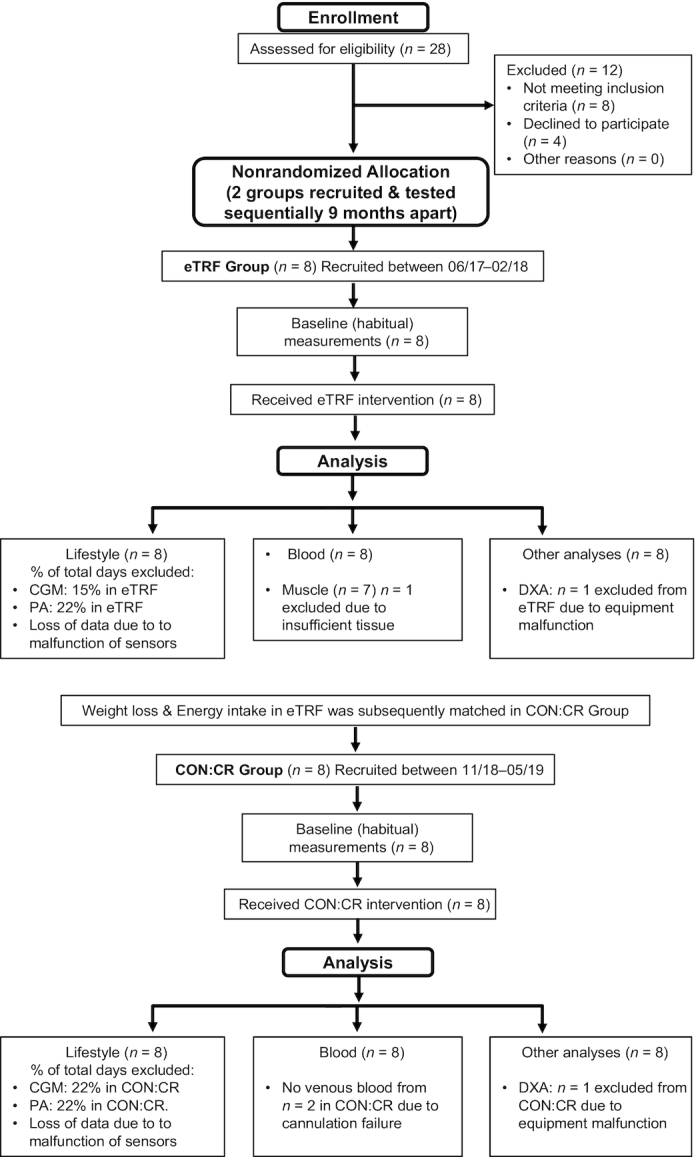
CONSORT flow diagram of study protocol. CGM, continuous glucose monitoring; CON:CR, control/caloric restriction; DXA, dual-energy X-ray absorptiometry; eTRF, early time-restricted feeding; PA, physical activity.

This study was approved by the University of Nottingham Faculty of Medicine and Health Sciences Research Ethics Committee (Ref. No. 19–1705) and performed at the David Greenfield Human Physiology Unit, University of Nottingham. It was registered at clinicaltrials.gov as NCT03969745 and met the regulations outlined in the Declaration of Helsinki. Informed and written consent was obtained from all individuals prior to enrollment in the study. A CONSORT flow diagram outlining the study protocol is displayed in **Figure 1**.

### Participants

This study was undertaken on 16 healthy young males [aged 23 ± 1 y; BMI (in kg·m^−2^) 24.0 ± 0.6; mean ± SEM]. Eligibility was assessed at a medical screening, which included anthropometric measurements, blood pressure assessment, a 12-lead electrocardiogram, and a blood sample for routine screening. Inclusion criteria included men aged between 18 and 35 y recruited from the university and general public, BMI between 18 and 27, and a moderately active PA level (PAL) between 1.6 and 1.99 (PAL = total energy expenditure/basal metabolic rate). Exclusion criteria included any metabolic, endocrine, or cardiovascular health condition; taking prescription medication that may influence cardiovascular and/or metabolic function; smoking; regular high alcohol consumption; irregular breakfast consumption (<5 d/wk); and an Eating Attitudes Test–26 score >20.

### Experimental design

Two experimental groups (matched for sex, age, BMI, and PA) completed a 1-wk baseline period to establish participants’ habitual dietary and PA patterns before they underwent a 2-wk dietary intervention [either eTRF or control/caloric restriction (CON:CR)]. Since imposing restrictions on the length of the daily EI window in free-living individuals consuming an ad libitum diet has been shown to lead to a reduction in EI (25), the eTRF study arm was completed first. This permitted weight loss and the macronutrient composition and caloric content of dietary intake in eTRF to be matched in the control group (CON:CR), which was recruited for separately 9 mo after completion of eTRF (Figure 1). Participants in both intervention groups were instructed to maintain their habitual PA patterns throughout the 2-wk intervention.

Initially, resting metabolic rate (RMR) was obtained for all participants following an overnight fast via indirect calorimetry as described below under Pre- and Post-intervention Main Experimental Trials. Participants in both groups also completed a modified incremental treadmill protocol with indirect calorimetry measurements (26) to improve the accuracy of free-living energy expenditure (EE) estimates obtained from a combined heart rate and accelerometer (Actiheart; CamNtech). Subjects were also fitted with a subcutaneous glucose monitor (Freestyle Libre; Abbott Diabetes Care), which recorded interstitial glucose concentrations every 15 min, and were instructed to record all EI using daily food diaries during a baseline week to establish the habitual PA and dietary patterns, respectively. CGM and food diaries were also recorded for the entire experimental intervention periods and used to verify adherence to each eating pattern.

Following the baseline week, participants visited the laboratory to assess their metabolic response to a carbohydrate (CHO) and protein liquid test meal (see Pre- and Post-intervention Main Experimental Trials below). From the next day, participants were either asked to restrict their daily EI window to between 08:00 and 16:00 (eTRF) or follow their habitual pattern without altering the temporal distribution of EI (CON:CR). In the former group, participants were encouraged to not consciously alter the type or quantity of food habitually consumed. In the CON:CR group, participants were prescribed dietary plans and provided with all food and beverages that matched the macronutrient composition (45% CHO, 35% fat, and 20% protein) and caloric content in eTRF. Participants in both groups were asked to restrict alcohol consumption to ≤1 unit per day throughout the intervention. Total daily EE for each participant was estimated using RMR and personalized PAL estimated from accelerometry and heart rate data. The deficit in EI in the CON:CR group was calculated based on the average involuntary reduction observed in the eTRF group and was scaled to the habitual total daily EE (as determined above). By using objective measurements of PA and body mass and composition throughout each intervention, any methodologic limitations of relying on food diaries for the estimation of dietary intake deficits were minimized. After 2 wk, participants in both groups visited the laboratory for a second main experimental trial to undergo identical follow-up metabolic testing.

### Pre- and post-intervention main experimental trials

**FIGURE 2:**
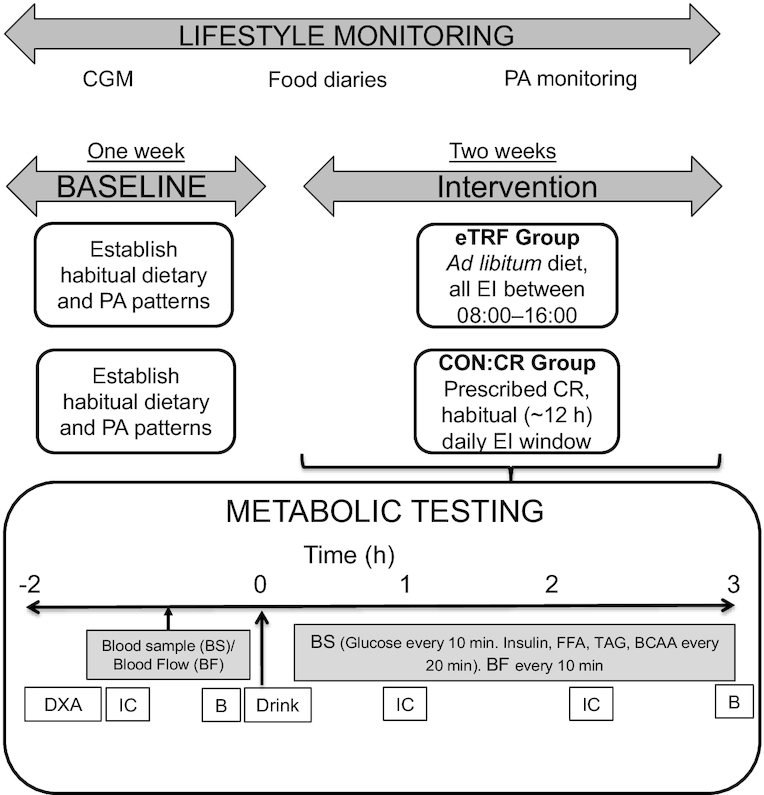
Schematic of experimental design and metabolic testing protocol. B, vastus lateralis biopsy; BF, brachial artery blood flow; BS, blood sampling; CGM, continuous glucose monitoring; CON:CR, control/caloric restriction; CR, caloric restriction; DXA, dual-energy X-ray absorptiometry; EI, energy intake; eTRF, early time-restricted feeding; IC, indirect calorimetry; PA, physical activity.

Metabolic measurements were undertaken before and for 180 min after consumption of a CHO and protein drink. On the day prior to each experimental, trial participants avoided alcohol consumption and strenuous exercise. On each occasion, an identical standardized evening meal was provided (45% CHO, 35% fat, 20% protein). Participants arrived at the laboratory the next day at 08:00 after a standardized overnight (12-h) fast. A schematic diagram of the experimental protocol followed is outlined in **Figure 2**. Upon arrival, subjects provided a urine sample before their body mass was recorded in light clothing. Following this, a DXA scan (Lunar Prodigy DXA; GE Medical Systems) was undertaken with subjects resting motionless in a supine position.

Subjects then rested semisupine in a bed while two retrograde cannulas were inserted, one guided by ultrasound (Aplio 300; Toshiba) into a deep-lying branch of an antecubital vein of the forearm and the other into a superficial hand vein of the contralateral arm. The latter was kept in a hand-warming unit maintained at 55°C to obtain arterialized venous blood samples that in our laboratory regularly achieve 96%–98% oxygen saturation of hemoglobin. Cannulas were kept patent using a saline drip, and samples were drawn simultaneously at baseline (fasted) and then every 10 min for 3 h following consumption of the liquid test meal. Brachial artery blood flow (BF_BA_; expressed in mL·min^−1^) was measured immediately after each blood sample using Doppler ultrasound. Rates of substrate (S) uptake [in μmol·L^−1^ for glucose and nmol·L^−1^ for branched-chain amino acids (BCAA)] across the forearm and glucose extraction (%) were calculated using the following equations:

(1) \begin{eqnarray*} {S_{uptake}} = \left( {{{[ S ]}_{arterialized}} - {\rm{\ }}{{[ S ]}_{venous}}} \right) \times B{F_{BA}} \end{eqnarray*}

(2) \begin{eqnarray*} && Glucose\ extraction\ \ \left( \% \right)\nonumber\\ &&\qquad = 100 \times \left( {\frac{{\left[ {arterialized\ glucose\ in\ mmol\cdot{L^{ - 1}}} \right]}}{{\left[ {venous\ glucose\ in\ mmol\cdot{L^{ - 1}}} \right]}}} \right )\end{eqnarray*}

### Blood flow and substrate uptake were standardized relative to lean forearm mass (in kg) determined by DXA

Vastus lateralis muscle biopsy specimens were obtained from 1 randomly assigned leg using the suction-modified Bergstrom technique (27, 28) before and 180 min after consumption of the drink in the eTRF intervention group only. Resting respiratory exchange ratio (RER), substrate (CHO and fat) oxidation rates, and EE were assessed before and 60 and 135 min after drink consumption via indirect calorimetry using a flow-based dilution canopy hood (Quark RMR; Cosmed) and values calculated using the equations of Frayn (29) and Weir (30). A second urine sample was collected at the end of the 3-h postprandial period. Both the baseline and postprandial urine samples were measured for volume and subsequently analyzed for the determination of urinary urea nitrogen using an enzymatic kinetic assay (Randox Cat# UR220), which allowed the calculation of nonprotein RER and rates of substrate oxidation.

The liquid test meal was standardized according to body weight (BW) and comprised 1 g·kgBW^−1^ dextrose, 0.4 g·kgBW^−1^ micellar casein protein (both from Bulk Powders), and 2 g cocoa powder (Cadbury Bournville) mixed into 4 mL·kgBW^−1^ water and accompanied by an additional 2 mL·kgBW^−1^ of water. Participants were allocated 10 min to fully consume the drinks, after which the 180-min postprandial period started.

### Blood analysis

For the determination of arterialized and deep venous blood glucose concentrations (fasted and every 10 min postprandial), aliquots of whole blood (0.5 mL) were rolled in sodium fluoride microtubes for 3 min before simultaneous analysis using a YSI 2300 (YSI). Then, 2-mL aliquots of blood samples were collected every 20 min into sodium heparin tubes containing EGTA-glutathione (15 µL) and EDTA tubes with aprotinin (100 µL) and were centrifuged immediately after collection at 4400 × *g* for 10 min at 4°C to obtain plasma. Another aliquot (3 mL) was left to coagulate in spray-coated silica and polymer gel tubes for 20–30 min before centrifugation at 4400 × *g* for 10 min at 4°C to obtain serum. All blood samples were stored at –80°C until analyses. Concentrations of serum triglyceride (TAG) and plasma free fatty acids (FFAs) were determined by enzymatic colorimetric assays on a clinical chemistry analyzer (ABX Pentra 400; Horiba). Serum insulin (cat. HI-014K; Merck Millipore) and total plasma ghrelin (cat. GHRT89HK; Merck Millipore) concentrations were determined using solid-phase ^125^I radioimmunoassay kits, which use the double antibody technique (31). Total plasma concentrations of BCAAs were determined spectrophotometrically using leucine dehydrogenase (32), as described in Wilhelmsen et al. (33). Due to the inevitable time elapsed between the 2 interventions, all analysis (other than plasma ghrelin) was undertaken separately for eTRF and CON:CR using appropriate controls to account for variability between assays.

### Skeletal muscle analysis

Muscle glycogen content was determined using a modified version of the protocol established by Harris (34). Pyruvate dehydrogenase complex (PDC) activity was determined using the method described by Constantin-Teodosiu (35). PDC-related acetyl-CoA formation rates were corrected for protein concentration using the Pierce bicinchoninic acid assay (ThermoFisher Scientific). Muscle BCAAs (leucine, isoleucine, and valine) and their respective keto acids (KAs; ketoisocaproic acid, 2-keto-3-methyl-valeric acid, and ketoisovaleric acid) were quantified by hydrophilic interaction and reverse-phase liquid chromatography (respectively) coupled to high-resolution mass spectrometry. Powdered muscle samples were vigorously vortexed for 5 min following the addition of 500 µL isopropanol (containing an appropriate amount of internal standard):1 mol·L^−1^ KH_2_PO_4_ buffer (1:1 vol:vol), and then for a further 5 min following the addition of 500 µL acetonitrile. Samples were then centrifuged for 20 min at 14,000 × *g* at 4°C. The supernatant was removed and evaporated to dryness under vacuum centrifuge, and samples were subsequently resuspended in 100 µL methanol:water (1:1 vol:vol) for LC-MS analyses. Absolute metabolite quantification was achieved using an isotopically (uniformly labeled ^13^C) internal standard method. Method validation in powdered skeletal muscle and a proxy matrix (7.5% BSA) showed excellent linearity (*R*^2^ > 0.99), accuracy and precision, and consistent levels of recovery across all metabolites.

### Outcomes

The primary outcomes of this study were the Matsuda index of (whole-body) insulin sensitivity (36) and forearm (skeletal muscle) glucose and BCAA uptake. Secondary outcomes were changes in free-living components of energy balance, body composition, and rates of substrate metabolism as assessed by indirect calorimetry. Exploratory outcomes included indices of glycemic variability under free-living conditions in both groups and muscle analysis of glycogen content, PDC activity, and BCAA and KA in response to feeding in the eTRF intervention group only to assess potential underlying mechanisms that can be exploited in future studies. The statistical power analysis indicated that 8 participants were required to detect a 15% improvement in postprandial whole-body insulin sensitivity (the primary outcome) with a power of 80% at a 5% significance level (37).

### Data handing and statistical analysis

Food diaries were kept by all participants for 6 full days during the baseline (habitual) period and for 13 d during each treatment period. Dietary analysis of food diaries was performed using Nutritics: (version 5.096; Nutritics Ltd, Ireland). Duration of the daily EI window was calculated as the time between the first and last entry in the food diary. CGMs were also recorded for 6 full days during the baseline (habitual) period and for 13 d during each treatment period. CGM data were shifted back to the nearest 15-min time point, and days with ≥20% missing data were excluded. Daily EE data from the Actiheart were recorded at 1-min epochs and analyzed using the “group calibration/individual HR + stress” model as per the manufacturer's instructions. This model limits the weighting of HR data to the algorithm used to estimate EE when the accelerometer detects little to no accompanying movement. Any days with ≥10% lost data or ≥22.5% recovered data were excluded from analyses. PALs were calculated as the ratio of daily total EE to resting metabolic rate. Furthermore, metabolic equivalent task (MET) values were used to estimate times spent at different intensities of PA.

All data analysis was performed in GraphPad Prism (version 7.04; GraphPad Software), except for mixed-design 3-factor ANOVA that was performed using SPSS Statistics (version 26; IBM Corp.). All data are presented as means ± SEMs. Pre- and post-intervention variables measured at a single time point within an experimental group were compared using paired 2-tailed *t* tests. Differences in metabolic variables between the 2 intervention groups (eTRF and CON:CR) were compared using independent samples 2-tailed *t* tests (for variables measured at baseline), a mixed-design 2-factor ANOVA (intervention group × pre-post trials) for variables measured at a single time point before (pre) and after (post) each intervention [including incremental areas under the curve (iAUCs)], or a mixed-design 3-factor ANOVA (intervention group × pre-post trials × sampling time) for variables measured at multiple time points before and after each intervention. Partial eta squared values (η^2^_p_) were calculated to illustrate the effect size for statistically significant intervention and interaction effects obtained when using ANOVA. Cohen's *d_s_* was used to quantify the effect size for statistically significant comparisons between groups when using independent samples *t* tests. All post hoc multiple comparisons following ANOVA that showed significant effects were undertaken using Bonferroni corrections. Statistical significance was accepted at *P* < 0.05.

## Results

### Subjects

There were no differences in age (22 ± 1 compared with 24 ± 2 y; *P* = 0.23), BMI (24.0 ± 1.0 compared with 23.8 ± 0.5; *P* = 0.99), PAL (1.71 ± 0.06 compared with 1.74 ± 0.05; *P* = 0.65), or HOMA-IR (1.29 ± 0.20 compared with 0.98 ± 0.13; *P* = 0.21) at baseline (habitual period) between the eTRF and CON:CR groups, respectively.

### Energy intake

The length of the daily EI window of participants’ habitual diet was similar before the eTRF (739 ± 15 min) and CON:CR (680 ± 27 min) interventions. This was shortened during eTRF (412 ± 16 min) compared with CON:CR (701 ± 22 min) [between-group difference (95% CI): 289 (231, 347) min; *P* = 0.00001; Cohen's *d_s_* = 5.3]. Self-reported daily EI was lower during eTRF compared with habitual EI (2318 ± 142 compared with 2722 ± 213 kcal; *P* = 0.01). Those self-reported time windows were verified by examining the pattern of glucose excursions using the 24-h CGM data. However, the relative energy contribution from CHO, fat, and protein was unchanged (post- compared with pre-eTRF; CHO, 44.7% ± 2.1% compared with 42.5% ± 1.9%, *P* = 0.47; fat, 34.9% ± 1.7% compared with 36.1% ± 1.1%, *P* = 0.59; protein, 20.0% ± 1.5% compared with 19.9% ± 1.2, *P* = 0.96), and this was carefully replicated in CON:CR.

### Physical activity energy expenditure

PAL values were similar between eTRF and CON:CR both before (1.71 ± 0.06 compared with 1.74 ± 0.05) and after (1.74 ± 0.08 compared with 1.77 ± 0.09) the interventions [between-group difference (95% CI): −0.03 (−0.21, 0.15)]. Similarly, there were no differences between intervention groups in daily time spent at light (1.5 ≤ METs < 3), moderate (3 ≤ METs < 6) or vigorous (≥6 METs) intensity of PA (**Supplemental Table 1**).

### Body weight and composition

There were no differences between CON:CR and eTRF in any of the body composition indices at baseline (preintervention). Body, lean, and fat mass responses to the interventions are reported in **Table 1** and were similar between eTRF and CON:CR [between-group differences (95% CI): body mass, −0.20 (−1.14, 0.73) kg; lean mass, 0.06 (−1.54, 1.65) kg; fat mass, −0.13 (−1.56, 1.31) kg; android fat, −0.04 (−0.13, 0.05) kg; gynoid fat, 0.07 (−0.16, 0.29) kg].

**TABLE 1:** Body mass and composition (assessed by DXA) before (pre) and after (post) 2 wk of eTRF or CON:CR^1^

Characteristic	Pre-CON:CR	Pre-eTRF	Post-CON:CR	Post-eTRF
Body mass, kg	77.68 ± 4.57	73.40 ± 2.97	76.44 ± 4.45**	72.36 ± 3.00**
Fat mass, kg	17.17 ± 3.38	12.82 ± 1.36	16.43 ± 3.38	12.20 ± 1.44
Lean mass, kg	57.42 ± 2.55	56.63 ± 2.86	56.95 ± 2.56	56.11 ± 3.11
Android fat, kg	1.58 ± 0.34	1.06 ± 0.13	1.48 ± 0.32**	0.98 ± 0.13*
Gynoid fat, kg	3.56 ± 0.64	2.65 ± 0.26	3.46 ± 0.64	2.47 ± 0.24*

^1^Data are means ± SEMs; *n* = 7 per group, except for body mass (*n* = 8). Mixed-design 2-factor ANOVA revealed no differences between CON:CR and eTRF in any of the indices but significant effects of time only on body mass (*P* = 0.0001), android fat (*P* = 0.0004), and gynoid fat (*P* = 0.02). Post hoc comparisons: *P = 0.04, **P = 0.01 from corresponding pre measurements. CON:CR, control/caloric restriction; eTRF, early time-restricted feeding.

### Continuous glucose monitoring

**FIGURE 3:**
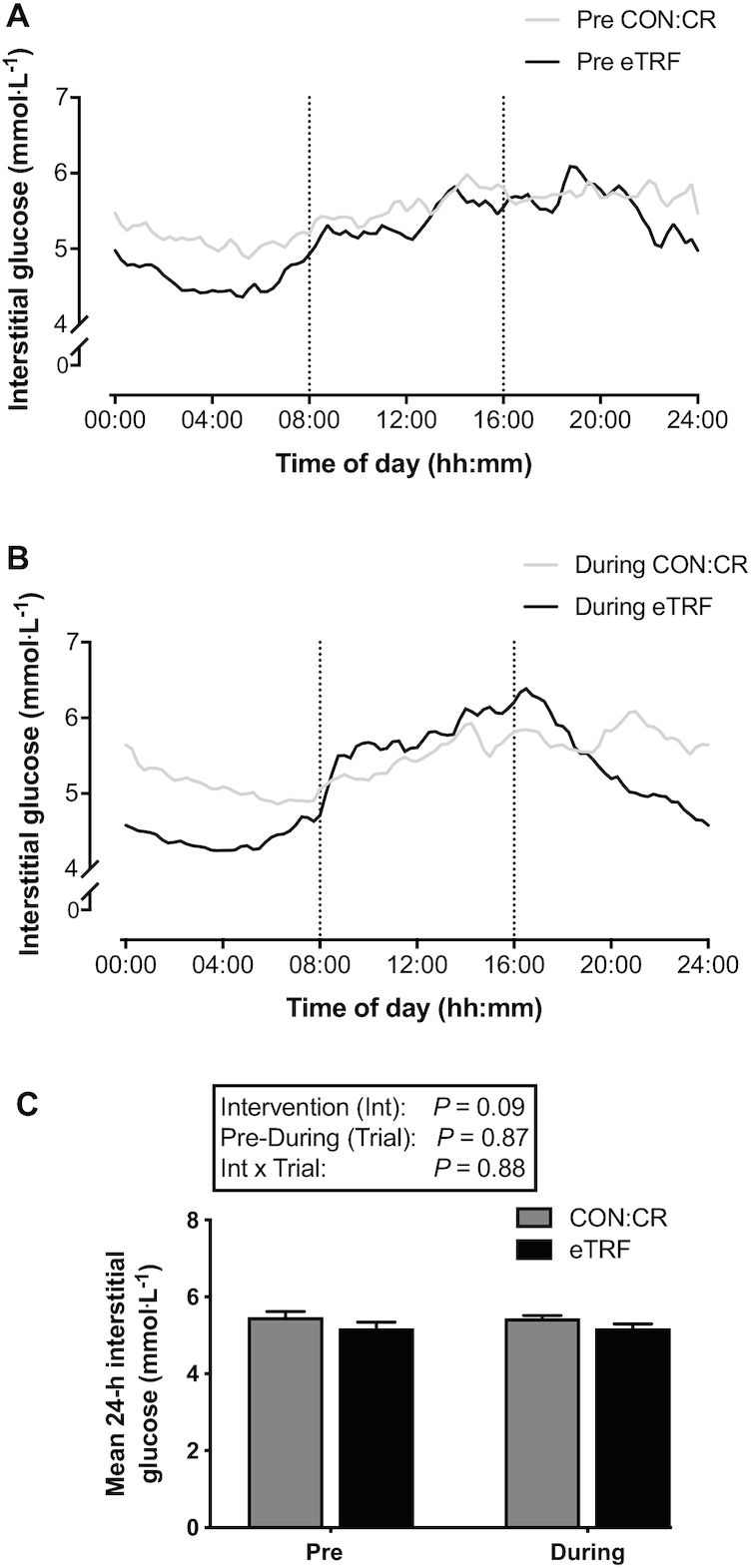
Diurnal interstitial glucose concentrations during eTRF and CON:CR. Data are means; *n* = 8 per group. The vertical dotted lines in (A) and (B) represent the start and end of the eating window during eTRF. CON:CR, control/caloric restriction; eTRF, early time-restricted feeding. Mixed-design 2-factor ANOVA (intervention group compared with pre-post trial) revealed no differences in any of the main effects or interaction between groups in mean 24-h interstitial glucose concentrations (C).

Diurnal interstitial glucose profiles before and during eTRF and CON:CR are displayed in **Figure 3**A and B. There were no significant differences in mean 24-h interstitial glucose concentrations between eTRF and CON:CR [between-group difference (95% CI): −0.28 (−0.61, 0.05) mmol·L^−1^] (Figure 3C). However, there was a difference between interventions in 24-h glycemic variability expressed as %CV (pre-eTRF 18.1 ± 1.0 compared with post-eTRF 19.7 ± 1.2; pre-CON:CR 14.2 ± 1.0 compared with post-CON:CR 13.4 ± 1.0), which was driven by higher values in eTRF than CON:CR both before and after the intervention [between-group difference (95% CI): 5.1% (2.4%, 7.8%); main intervention effect *P* = 0.001; η^2^_p_ = 0.55].

When separate subanalysis was performed on the 08:00 to 20:00 and 20:00 to 08:00 time windows, there were lower mean glucose concentrations only in the latter window pre- and post-eTRF compared with CON:CR [between-group difference (95% CI): −0.58 (−0.92, −0.23) mmol·L^−1^; main intervention effect *P* = 0.003; η^2^_p_ = 0.47]. Furthermore, %CV was similar between interventions during 20:00 to 08:00 but higher during 08:00 to 20:00 [between-group difference (95% CI): 4.3% (1.7%, 6.8%); main intervention effect *P* = 0.003; η^2^_p_ = 0.48] in eTRF compared with CON:CR (**Table 2**).

**TABLE 2:** Continuous glucose monitoring indices during 1 wk of habitual diet (pre) and during 2 wk of eTRF or CON:CR^1^

Characteristic	Pre-CON:CR	Pre-eTRF	During CON:CR	During eTRF
Mean glucose, mmol·L^−^^1^				
08:00 to 20:00	5.58 ± 0.14	5.49 ± 0.19	5.53 ± 0.09	5.75 ± 0.13
20:00 to 08:00	5.34 ± 0.16	4.93 ± 0.18*	5.33 ± 0.07	4.58 ± 0.12***
Glucose variability, %CV				
08:00 to 20:00	13.6 ± 1.1	17.8 ± 0.7**	14.2 ± 1.2	18.6 ± 1.1**
20:00 to 08:00	13.9 ± 1.0	16.7 ± 1.4	14.0 ± 0.8	13.5 ± 1.4

^1^Data are means ± SEMs; *n* = 8 for both CON:CR and eTRF. Separate mixed-design 2-factor ANOVA was performed for the 08:00 to 20:00 and 20:00 to 08:00 time windows that were not directly compared. The analysis revealed an intervention (group) effect (*P* = 0.003) between CON:CR and eTRF for mean glucose during 20:00 to 08:00 only (post hoc: *P = 0.02 and ***P = 0.001 from corresponding CON:CR) and an intervention effect (*P* = 0.003) between CON:CR and eTRF for %CV during 08:00 to 20:00 only (post hoc: **P = 0.01 from corresponding CON:CR). CON:CR, control/caloric restriction; eTRF, early time-restricted feeding.

### Fasting measurements

There were no differences in fasting arterialized blood glucose [between-group difference (95% CI): −0.01 (−0.32, 0.30) mmol·L^−1^], plasma BCAAs [between-group difference (95% CI): 46 (−14, 105) μmol·L^−1^], serum TAG [between-group difference (95% CI): −0.05 (−0.25, 0.35) mmol·L^−1^], or plasma FFA [between-group difference (95% CI): −0.02 (−0.21, 0.18) mmol·L^−1^] both before and after 2 wk of eTRF or CON:CR (**Table 3**). However, fasting serum insulin [between-group difference (95% CI): 29 (8, 49) ρmol·L^−1^; main intervention effect *P* = 0.01; η^2^_p_ = 0.39] and plasma ghrelin [between-group difference (95% CI): 66 (20, 112) ρmol·L^−1^; main intervention effect *P* = 0.01; η^2^_p_ = 0.40] concentrations were lower pre- and post-CON:CR, respectively, compared with the eTRF. Resting metabolic rate was unchanged in response to eTRF (pre 1829 ± 87 compared with post 1812 ± 62 kcal·d^−1^) and CON:CR (pre 1937 ± 91 compared with post 1857 ± 70 kcal·d^−1^) with no differences observed between interventions.

**TABLE 3:** Fasted arterialized metabolites before (pre) and after (post) 2 wk of eTRF or CON:CR^1^

Characteristic	Pre-CON:CR	Pre-eTRF	Post-CON:CR	Post-eTRF
Glucose, mmol·L^−1^	4.13 ± 0.09	4.03 ± 0.08	4.00 ± 0.13	4.08 ± 0.17
Serum insulin, ρmol·L^−1^	37 ± 5**	74 ± 12	43 ± 5	63 ± 7
Serum TAG, mmol·L^−1^	0.72 ± 0.10	0.81 ± 0.12	0.73 ± 0.10	0.73 ± 0.09
Plasma FFA, mmol·L^−1^	0.54 ± 0.04	0.51 ± 0.09	0.58 ± 0.09	0.58 ± 0.06
Plasma BCAA, μmol·L^−1^	207 ± 20	263 ± 18	222 ± 21	257 ± 23
Plasma total ghrelin, ρmol·L^−1^	229 ± 19	282 ± 15	203 ± 21**	281 ± 15

^1^Data are means ± SEMs; *n* = 8 per group. Mixed-design 2-factor ANOVA revealed an intervention (group) effect (*P* = 0.01) for both insulin and ghrelin (post hoc **P = 0.01 from corresponding eTRF time point). BCAA, branched-chain amino acid; CON:CR, control/caloric restriction; eTRF, early time-restricted feeding; FFA, free fatty acid; TAG, triglyceride.

### RER and substrate oxidation rates

Significant interaction effects (intervention group × pre-post trial) were observed on fasting nonprotein RER [between-group difference (95% CI): −0.07 (−0.13, −0.01); interaction effect *P* = 0.03; η^2^_p_ = 0.28] and rates of CHO oxidation [between-group difference (95% CI): −0.08 (−0.16, −0.01) g·min^−1^; interaction effect *P* = 0.04; η^2^_p_ = 0.26] but not fat oxidation [between-group difference (95% CI): −0.01 (−0.03, 0.02) g·min^−1^] (**Table 4**). However, there were no differences in postprandial rates of nonprotein RER [between-group difference (95% CI): −0.01 (−0.05, 0.02)], CHO oxidation [between-group difference (95% CI): −0.03 (−0.08, 0.02) g·min^−1^], or fat oxidation [between-group difference (95% CI): −0.001 (−0.023, 0.020) g·min^−1^] between eTRF and CON:CR (Table 4).

**TABLE 4:** RER and rates of substrate metabolism before (fasted) and in response to postprandial (PP) consumption of a CHO and protein drink before (pre) and after (post) eTRF and CON:CR^1^

	Pre-CON:CR		Pre-eTRF		Post-CON:CR		Post-eTRF	
Characteristic	Fasted	PP	Fasted	PP	Fasted	PP	Fasted	PP
RER	0.79 ± 0.03	0.87 ± 0.03	0.75 ± 0.02	0.85 ± 0.02	0.75 ± 0.01	0.87 ± 0.01	0.77 ± 0.02	0.85 ± 0.01
CHOox, g·min^–1^	0.11 ± 0.03	0.21 ± 0.03	0.04 ± 0.02	0.18 ± 0.02	0.06 ± 0.02	0.20 ± 0.02	0.08 ± 0.02	0.16 ± 0.02
FATox, g·min^–1^	0.10 ± 0.02	0.07 ± 0.02	0.11 ± 0.01	0.06 ± 0.01	0.11 ± 0.01	0.06 ± 0.01	0.09 ± 0.01	0.06 ± 0.01

^1^Data are presented as means ± SEMs; *n* = 8 per group. All PP values refer to the average of measurements throughout the 3-h postprandial period. Separate mixed-design 2-factor ANOVA (intervention group × pre-post trials) for fasted and PP data revealed significant interactions for fasted RER (*P* = 0.03) and CHOox (*P* = 0.04) but no FATox (*P* = 0.08), whereas no differences were observed between eTRF and CON:CR in any of the PP indices. CHOox, carbohydrate oxidation rates; CON:CR, control/caloric restriction; eTRF, early time-restricted feeding; FATox, fat oxidation rates; PP, 3-h postprandial period following the test meal consumption; RER, nonprotein respiratory exchange ratio.

### Plasma FFA and serum TAG

**FIGURE 4:**
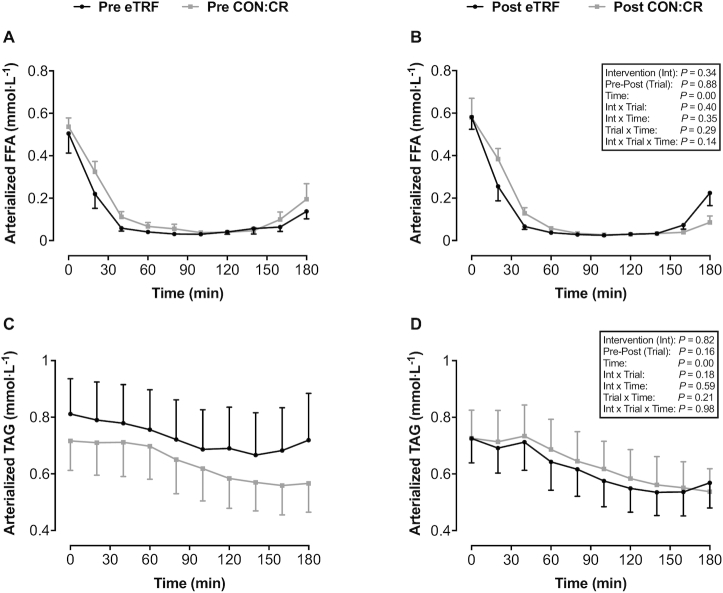
(A, B) Arterialized plasma FFA and (C, D) serum TAG in response to consumption of a liquid test meal before (pre) and after (post) 2 wk of dietary intervention. All data are means ± SEMs; *n* = 8 per group. *P* values displayed in text boxes refer to mixed-design 3-factor ANOVA (intervention group × pre-post trial × sampling time) performed for the entire postprandial period. CON:CR, control/caloric restriction; eTRF, early time-restricted feeding; FFA, free fatty acid; TAG, triglyceride.

In both eTRF and CON:CR, ingestion of the CHO and protein drink rapidly suppressed circulating plasma FFA (main time effect *P* = 0.0001; η^2^_p_ = 0.84), but there were no significant intervention or interaction effects (**Figure 4**A and B). Similarly, serum TAG concentrations were lower after ingestion of the CHO and protein drink (main time effect *P* = 0.0001; η^2^_p_ = 0.63) compared with fasting values, but no differences were shown between eTRF and CON:CR (Figure 4C and D).

### Blood glucose, serum insulin, and whole-body insulin sensitivity

**FIGURE 5:**
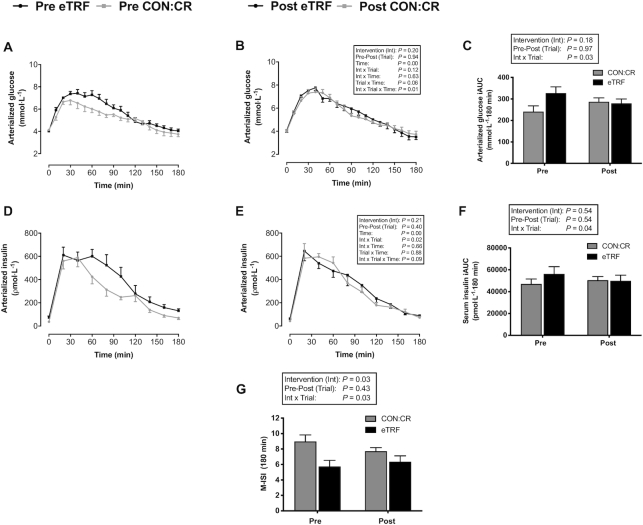
Indices of whole-body insulin sensitivity: (A, B) arterialized blood glucose, (C) iAUC for glucose, (D, E) arterialized serum insulin, (F) iAUC for insulin, and (G) Matsuda index of insulin sensitivity in response to consumption of a liquid test meal before (pre) and after (post) 2 wk of dietary intervention. All data are means ± SEMs; *n* = 8 per group. *P* values displayed in text boxes refer to mixed-design 3-factor ANOVA (intervention group × pre-post trial × sampling time) performed for the entire postprandial period (A, B, D, and E) and mixed-design 2-factor ANOVA (intervention group × pre-post trial) performed for iAUC data (C, F). CON:CR, control/caloric restriction; eTRF, early time-restricted feeding; iAUC, incremental area under the curve; M-ISI, Matsuda index of insulin sensitivity.

In both interventions, circulating glucose and insulin concentrations increased in response to ingestion of the CHO and protein drink. However, a significant interaction effect (intervention group × pre-post trial × sampling time) on circulating glucose was observed (*P* = 0.01; η^2^_p_ = 0.13), with values increasing post-CON:CR and decreasing post-eTRF compared with their respective preintervention trials (**Figure 5**A and B). Accordingly, an interaction effect (intervention group × pre-post trial) was also observed between intervention groups for the glucose iAUC [between-group difference (95% CI): 93 (11, 176) mmol·L^−1^·180 min; main interaction effect *P* = 0.03; η^2^_p_ = 0.29], which decreased after eTRF and increased after CON:CR compared with their respective preintervention concentrations (Figure 5C).

Significant interaction effects (intervention group × pre-post trial) were observed for both the serum insulin concentrations (*P* = 0.02; η^2^_p_ = 0.34) and iAUC [between-group difference (95% CI): 9697 (248, 19,146) ρmol·L^−1^·180 min; main interaction effect *P* = 0.04; η^2^_p_ = 0.26] across the entire postprandial period, with values decreasing after eTRF and increasing after CON:CR compared with their respective preintervention concentrations (Figure 5D–F).

The Matsuda insulin sensitivity index (M-ISI), a composite index of hepatic and peripheral insulin sensitivity, improved after 2 wk of eTRF but declined in the CON:CR group as indicated by a significant interaction effect [between-group difference (95% CI): 1.89 (0.18, 3.60); intervention group × pre-post trial interaction effect *P* = 0.03; η^2^_p_ = 0.29] shown in Figure 5G.

### Skeletal muscle insulin sensitivity

**FIGURE 6:**
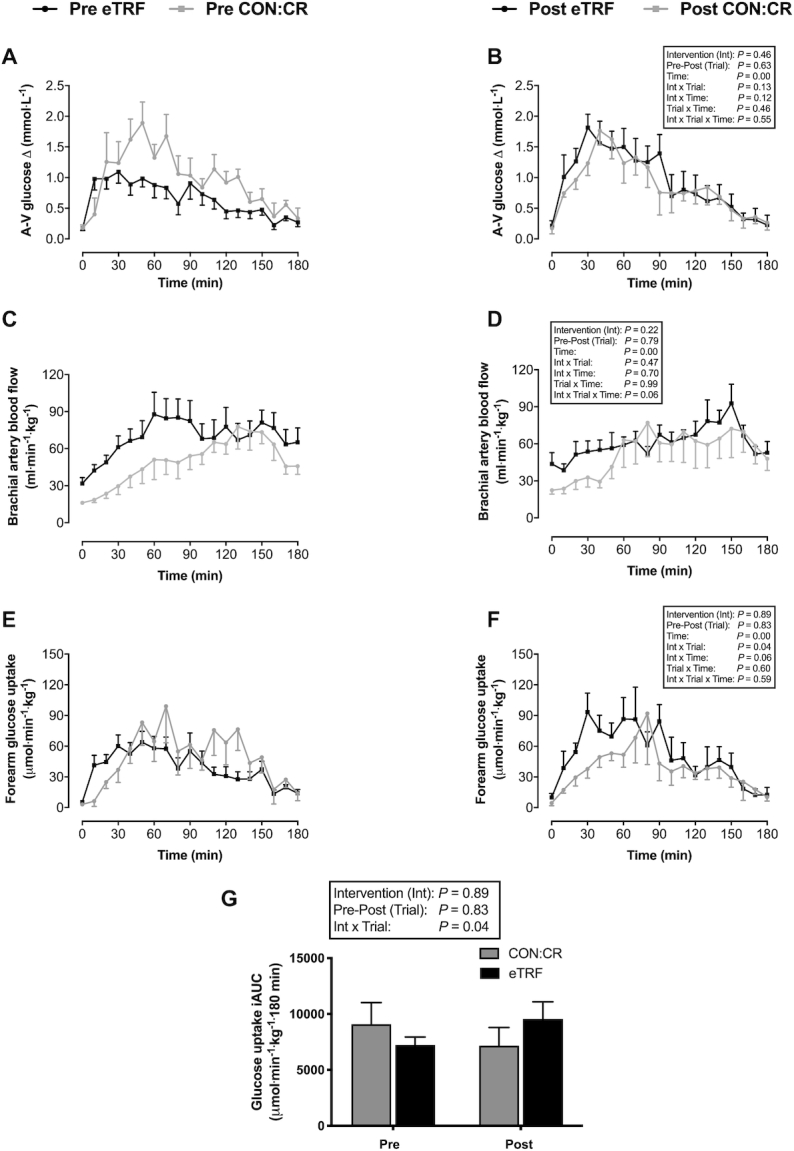
(A, B) Arteriovenous glucose differences, (C, D) brachial artery blood flow, and (E–G) forearm (skeletal muscle) glucose uptake in response to consumption of a liquid test meal before (pre) and after (post) 2 wk of dietary intervention. Data are means ± SEMs. *n* = 8 for eTRF and n = 6 for CON:CR. Blood flow and glucose uptake are standardized relative to lean forearm mass (in kg) determined by DXA. *P* values displayed in text boxes refer to mixed-design 3-factor ANOVA (intervention group × pre-post trial × sampling time) performed for the entire postprandial period (A–F) and mixed-design 2-factor ANOVA (intervention group × pre-post trial) performed for iAUC data (G). CON:CR, control/caloric restriction; eTRF, early time-restricted feeding.

In both interventions, brachial artery blood flow and arteriovenous (AV) glucose differences increased in response to ingestion of the CHO and protein drink, but no significant intervention or interaction effects were observed (**Figure 6**A–D). Brachial blood flow was similar post-CON:CR but tended to be lower post-eTRF compared with preintervention trials (interaction effect for intervention group × pre-post trial × sampling time, *P* = 0.06; η^2^_p_ = 0.12) (Figure 6C and D).

Significant interaction effects (intervention group × pre-post trial) were observed for the forearm (muscle) glucose uptake (*P* = 0.04; η^2^_p_ = 0.30) and iAUC [between-group difference (95% CI): 4266 (261, 8270) μmol·min^−1^·kg^−1^·180 min; *P* = 0.04; η^2^_p_ = 0.31] across the entire postprandial period, which increased post-eTRF and decreased post-CON:CR compared with their respective preintervention concentrations (Figure 6E–G). Glucose extraction was unaffected by eTRF (pre 11% ± 2% compared with post 15% ± 3%) and CON:CR (pre 17% ± 2% compared with post 15% ± 3%) with no significant difference observed between interventions [between-group difference (95% CI): −7% (−15%, 2%); interaction effect *P* = 0.10; η^2^_p_ = 0.21].

### Plasma BCAA concentrations

**FIGURE 7:**
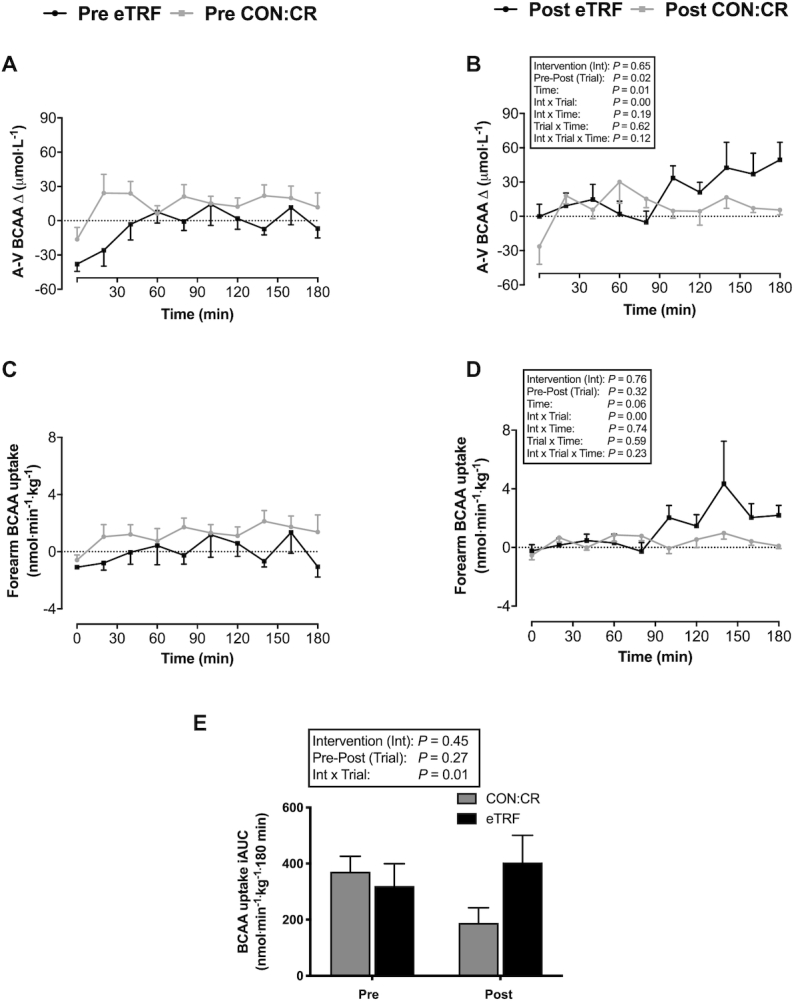
(A, B) Arteriovenous plasma BCAA differences and (C–E) forearm (skeletal muscle) BCAA uptake in response to consumption of a liquid test meal before (pre) and after (post) 2 wk of dietary intervention. Data are means ± SEMs. *n* = 8 for eTRF and *n* = 6 for CON:CR. Forearm BCAA uptake is expressed relative to lean forearm mass (in kg) determined by DXA. *P* values displayed in text boxes refer to mixed-design 3-factor ANOVA (intervention group × pre-post trial × sampling time) performed for the entire postprandial period (A–D) and mixed-design 2-factor ANOVA (intervention group × pre-post trial) performed for incremental area under the curve data (E). BCAA, branched-chain amino acids; CON:CR, control/caloric restriction; eTRF, early time-restricted feeding.

In both intervention groups, AV BCAA differences increased after ingestion of the CHO and protein drink. However, an interaction effect (intervention group × pre-post trial) was observed, with higher AV BCAA differences post-eTRF and lower values post-CON:CR compared with their respective preintervention trials (*P* = 0.001; η^2^_p_ = 0.59; **Figure 7**A and B). A similar pattern of interaction (intervention group × pre-post trial) was shown in BCAA forearm (muscle) uptake (*P* = 0.01; η^2^_p_ = 0.64) and iAUC [between-group difference (95% CI): 266 (77, 455) nmol·min^−1^·kg^−1^·180 min; *P* = 0.01; η^2^_p_ = 0.44] across the entire postprandial period, which increased post-eTRF and decreased post-CON:CR compared with preintervention trials (Figure 7C–E).

### Muscle glycogen, PDC activity, and BCCA content

We next sought to explore the intramuscular fate of the increased skeletal muscle glucose and BCAA uptake after eTRF. Muscle glycogen content, an index of nonoxidative glucose disposal, was similar in the fasted condition and remained unchanged [within-group difference (95% CI): 21 (−65, 108) mmol glycosyl units·kg^−1^ dry weight] 180 min postconsumption of the CHO and protein drink (**Table 5**). PDC activity, the rate-limiting step in muscle glucose oxidation, increased after ingestion of the CHO and protein drink [fast-fed difference (95% CI): 0.94 (0.53, 1.36) nmol·mg protein^−1^·min^−1^; *P* = 0.002; η^2^_p_ = 0.84] but no difference was found between pre- and post-eTRF [within-group difference (95% CI): 0.30 (−1.43, 2.04) nmol·mg protein^−1^·min^−1^]. Fasting and postprandial muscle BCAA (sum of leucine, isoleucine, and valine) concentrations were not significantly different post-eTRF compared with pre-eTRF [within-group difference (95% CI): 301 (−62, 664) µmol·kg^−1^ dry weight; interaction effect *P* = 0.10; η^2^_p_ = 0.21] (Table 5). Fasting and postprandial muscle branched-chain KA (sum of ketoisocaproic, 2-keto-3-methyl-valeric, and ketoisovaleric acids) concentrations were unaffected by the eTRF intervention [within-group difference (95% CI): 6 (−28, 16) µmol·kg^−1^ dry weight] (Table 5).

**TABLE 5:** Skeletal muscle analysis before (pre) and 2 wk after (post) eTRF^1^

	Pre-eTRF		Post-eTRF	
Characteristic	0 min	180 min	0 min	180 min
BCAA, µmol·kgDW^−1^	2059 ± 150	1839 ± 122	1934 ± 132	2015 ± 171
KA, µmol·kgDW^−1^	56.4 ± 6.2	44.6 ± 8.3	58.0 ± 8.2	55.7 ± 12.0
Glycogen, mmol glycosyl units·kgDW^−1^	359 ± 25	360 ± 36	382 ± 48	380 ± 43
PDC activity, nmol·mg protein^−1^· min^−1^	4.17 ± 0.64	4.41 ± 0.61**	3.77 ± 0.34	5.42 ± 0.74**

^1^Data are means ± SEMs; *n* = 7. Mixed-design 2-factor ANOVA revealed no intervention or interaction effects for muscle BCAA (*P* = 0.89 and *P* = 0.10, respectively), KA (*P* = 0.53 and *P* = 0.37, respectively), glycogen (*P* = 0.57 and *P* = 0.96, respectively), or PDC (*P* = 0.69 and *P* = 0.30, respectively). A time effect (**P = 0.002 from respective 0 min) was observed for PDC only. BCAA, branched-chain amino acid (sum of leucine, isoleucine, and valine); DW, dry weight; eTRF, early time-restricted feeding; KA, branched-chain keto acids (sum of ketoisocaproic, 2-keto-3-methyl-valeric, and ketoisovaleric acids); PDC, pyruvate dehydrogenase complex.

## Discussion

The present study shows that under free-living conditions, 2 wk of eTRF improves whole-body insulin sensitivity and increases postprandial skeletal muscle nutrient (glucose and amino acids) uptake in healthy young men. Importantly, our results suggest these beneficial effects are independent of the changes in body composition and energy balance elicited by a reduction in free-living EI during eTRF. Moreover, unlike previous research (15), they represent chronic adaptations to eTRF, rather than the acute effect of the last bout of prolonged overnight fast, as the duration of fast prior to metabolic testing was standardized to habitual levels.

The improvement in whole-body insulin sensitivity is evidenced by the lower postprandial glycemic and insulinemic responses and higher M-ISI in response to consumption of the CHO and protein drink in eTRF compared with CON:CR. This extends recent findings on overweight men with prediabetes (15) to a healthier population. In contrast, no reductions in fasting insulin were shown, which is in line with previous observations made in males with obesity after a standardized fast (13). However, 8 wk of TRF have previously shown to lower fasting insulin in healthy resistance-trained males (6), suggesting 2 wk may have been insufficient to detect a difference in fasting insulin.

Incorporating protein and dextrose into the oral liquid test challenge permitted simultaneous insight into postprandial skeletal muscle glucose and BCAA uptake, as indices of muscle insulin and anabolic sensitivity, respectively. As no significant differences in blood flow to the muscle were observed between interventions, the elevated glucose uptake in the postprandial period of the eTRF trial (which was particularly prominent in the initial 90 min of it) could be accounted for by improved efficiency of glucose extraction. This is interesting, considering postprandial insulin concentrations were lower in eTRF compared with CON:CR.

The increased skeletal muscle glucose uptake after eTRF, compared with CON:CR, was not accompanied by higher whole-body postprandial CHO oxidation rates. Muscle glycogen content was also unchanged, suggesting nonoxidative glucose disposal was similar. Muscle PDC activation, the rate-limiting step in muscle glucose oxidation, was similar in the basal (fasted) condition and increased in response to consumption of the liquid test meal in both trials. More pronounced differences in PDC activation, and hence muscle glucose oxidation, may have been evident if the postprandial muscle biopsy was obtained earlier than 180 min, when glucose uptake and insulin concentrations were elevated.

Forearm BCAA uptake also increased in response to feeding after 2 wk of eTRF compared with CON:CR, which could be an adaptive mechanism to potentiate the anabolic response to protein ingestion as the body adapts to a shorter EI window. This may partially explain previous observations that TRF promotes improved retention of lean body mass during weight loss in response to resistance training (6), although this was not replicated in this study presumably due to its short-term nature and limited weight loss incurred. Increased uptake of plasma BCAA by skeletal muscle after eTRF in the present study is supported by our data showing a trend (*P* = 0.10) for increased intramuscular BCAA content after feeding. The metabolic fate (synthesis or oxidation) of these BCAAs remains unknown, although we observed no changes in their keto acids after feeding, which suggests a potential anabolic response. Future studies should address the efficacy of eTRF as a nutritional strategy to better preserve the protein synthetic response to a protein-rich meal and hence lean body mass under conditions of weight loss using stable isotope tracers.

Both prolonging the daily fast and shifting food intake to earlier in the day likely contributed to the underlying metabolic improvements after eTRF. Diurnal metabolic responses to nutrients are influenced by the circadian system, and glucose tolerance is higher earlier in the day (19–22). Although different EI windows (early compared with late TRF) were not directly compared in the present study, we postulate that chronically shifting nutrient intake to earlier in the day may have beneficial metabolic effects (15, 23–25). Although similar weight loss was induced by both interventions in the present study, there were no obvious metabolic benefits associated with the CON:CR group. The reasons are unclear but could relate to the short-term nature and limited body weight loss incurred in young physically active individuals that is unlikely to alter metabolic health.

Adherence to the eTRF protocol was high, with only 1 reported eating event outside of the stipulated EI window. This is further supported by the pronounced differences in glycemic profiles between CON:CR and eTRF, with lower mean glucose values evident between 20:00 and 08:00 (reflecting the longer overnight fast) and higher variability during 08:00 and 20:00 in eTRF (reflecting the shorter EI window). To our knowledge, only 1 other study (13) has compared free-living glucose concentrations during a habitual diet and eTRF. They also found no change in mean 24-h glucose but a reduction in fasting glucose concentration, defined as the time between 4 h after consumption of the last meal of the day until the time of the first meal of the next day. These findings are broadly comparable to the 20:00 to 08:00 fasting window used in the present study and in agreement with our findings of lower mean glucose values during that period in eTRF.

In line with previous research, imposing restrictions on the length of the daily EI window in a free-living setting leads to an involuntary reduction in EI (9, 25, 38). In the present study, this was estimated to be ∼400 kcal·d^−1^ and, in the absence of changes in objectively quantified PA levels, led to a negative energy balance that resulted in a mean body mass loss of 1.04 ± 0.25 kg over the 2-wk eTRF intervention period. Previous studies objectively measuring PA during TRF showed no changes but were conducted on adults who were overweight/obese, with low baseline PA levels (13, 38). The present study extends these findings to a more physically active population. This contrasts with reductions in PA observed during Ramadan (39, 40) and likely reflects the chronobiologically opposing EI windows used.

A major strength of this study is the comparable weight loss elicited in a control group matched for age, BMI, and PA. This was achieved by prescribing a diet matched for macronutrient composition, and importantly, participants did not alter their temporal distribution of EI during CON:CR. Together, these results suggest that the metabolic improvements observed after eTRF are independent of the energy imbalance and small but significant amount of weight lost elicited by a reduction in free-living EI. One limitation of the present study is that only healthy men were recruited, and there may be sex-based differences in responses to intermittent fasting (41). Other limitations include the short-term study duration and the use of self-reported energy intake data to prescribe an energy-matched diet for the CON:CR group. However, by using objective measurements of PA and body composition, any methodologic limitations of relying on food diaries for the estimation of dietary intake deficits were minimized. Those measurements also confirmed that the participants complied with the requirements of the study, including the voluntary energy deficit imposed on the participants in the CON:CR group.

In summary, the present study highlights skeletal muscle as an important tissue modulating the beneficial effects of eTRF on postprandial insulin and anabolic sensitivity in healthy men. Although improvements in insulin sensitivity are likely more relevant to clinical populations at an increased susceptibility to metabolic disease(s), even in healthy individuals, insulin sensitivity is an independent predictor of future cardiovascular disease (42, 43). Our results suggest that eTRF would lead to a natural reduction to EI and augment the insulin-sensitizing effect of the accompanying weight loss. This suggests eTRF has potential applications as an alternative to calorie counting and may provide a simplistic, accessible dietary intervention. However, larger-scale research studies are necessary to address its feasibility in the longer term. While research suggests eTRF may confer additional metabolic benefits over other intermittent fasting approaches, direct comparisons between different eating windows are necessary to guide practical dietary strategies.

## Supplementary Material

nqaa192_Supplemental_File

## Notes

Robert Jones and Pardeep Pabla, equal contribution.

Sources of support: RJ and PP funded by Biotechnology and Biological Sciences Research Council (BBSRC) Doctoral Training Partnership.

Data described in the manuscript, code book, and analytic code will be made available upon request.

Supplemental Table 1 is available from the “Supplementary data” link in the online posting of the article and from the same link in the online table of contents at https://academic.oup.com/ajcn/.

Abbreviations used: AV, arteriovenous; BCAA, branched-chain amino acid; BF_BA_, brachial artery blood flow; BW, body weight; CGM, continuous glucose monitor; CHO, carbohydrate; CON:CR, control/caloric restriction; EE, energy expenditure; EI, energy intake; eTRF, early time-restricted feeding; FFA, free fatty acid; iAUC, incremental area under the curve; KA, keto acid; MET, metabolic equivalent task; M-ISI, Matsuda insulin sensitivity index; PA, physical activity; PAL, physical activity level; PDC, pyruvate dehydrogenase complex; RER, respiratory exchange ratio; RMR, resting metabolic rate; S, substrate; TAG, triglyceride; TRF, time-restricted feeding.
