# Supplementary material for: Two weeks of early time-restricted feeding (eTRF) improves skeletal muscle insulin and anabolic sensitivity in healthy men
Source: Am J Clin Nutr. 2020 Jul 30;112(4):1015–28. doi: 10.1093/ajcn/nqaa192 (PMC7528549; doi:10.1093/ajcn/nqaa192)
Supplement: nqaa192_Supplemental_File [file nqaa192_supplemental_file.docx]

**On-line Supplementary Table**

**Authors:** Jones et al

**Title:** Two weeks of early time restricted feeding (eTRF) improves skeletal muscle insulin and anabolic sensitivity in healthy men

**Supplementary Table 1. Daily time (min) spent at different physical activity**

**intensities.**

|  | Pre CON:CR | Pre eTRF | Post CON:CR | Post eTRF |
| --- | --- | --- | --- | --- |
| Light (1.5 ≤ METs < 3) | 187 ± 21 | 182 ± 14 | 180 ± 25 | 189 ± 22 |
| Moderate (3 ≤ METs < 6) | 113 ± 18 | 118 ± 15 | 133 ± 25 | 118 ± 15 |
| Vigorous (≥ 6 METs) | 14 ± 3 | 13 ± 4 | 13 ± 2 | 20 ± 5 |
| Data is mean ± SEM; n=8 per group. CON:CR = control:caloric restriction; eTRF = early time-restricted feeding; METs = metabolic equivalent of task. Mixed design 2-way ANOVA revealed no intervention or interaction effects for any of the measured variables. | | | | |
